# Enhanced Expression of IGFBP-3 Reduces Radiosensitivity and Is Associated with Poor Prognosis in Oral Squamous Cell Carcinoma

**DOI:** 10.3390/cancers12020494

**Published:** 2020-02-20

**Authors:** Junki Sakata, Akiyuki Hirosue, Ryoji Yoshida, Yuichiro Matsuoka, Kenta Kawahara, Hidetaka Arita, Hikaru Nakashima, Tatsuro Yamamoto, Masashi Nagata, Sho Kawaguchi, Shunsuke Gohara, Yuka Nagao, Keisuke Yamana, Ryo Toya, Ryuji Murakami, Yoshikazu Kuwahara, Manabu Fukumoto, Hideki Nakayama

**Affiliations:** 1 Department of Oral and Maxillofacial Surgery, Faculty of Life Sciences, Kumamoto University, Kumamoto 860-8556, Japan; jsakata@kuh.kumamoto-u.ac.jp (J.S.); ryoshida@kumamoto-u.ac.jp (R.Y.); yu6600@kuh.kumamoto-u.ac.jp (Y.M.); karapote@kuh.kumamoto-u.ac.jp (K.K.); haritah1@aih-net.com (H.A.); hikarun@kuh.kumamoto-u.ac.jp (H.N.); tatsuro.yamamoto@jfcr.or.jp (T.Y.); nagatama0213@kuh.kumamoto-u.ac.jp (M.N.); idd09017m@kuh.kumamoto-u.ac.jp (S.K.); shun2707@kuh.kumamoto-u.ac.jp (S.G.); dd09034w@kuh.kumamoto-u.ac.jp (Y.N.); idd09053y@kuh.kumamoto-u.ac.jp (K.Y.); 2 Department of Radiation Oncology, Kumamoto University Hospital, Kumamoto 860-8556, Japan; ryo108@kumamoto-u.ac.jp; 3 Department of Medical Imaging, Faculty of Life Sciences, Kumamoto University, Kumamoto 862-0976, Japan; murakami@kumamoto-u.ac.jp; 4 Radiation Biology and Medicine, Faculty of Medicine, Tohoku Medical and Pharmaceutical University, 4-4-1, Komatsushima, Aoba, Sendai, Miyagi 981-8558, Japan; y-kuwahara@umin.ac.jp; 5 Department of Molecular Pathology, Tokyo Medical University, 6-1-1, Shinjuku, Shinjuku, Tokyo 160-8402, Japan; manabu.fukumoto.a8@tohoku.ac.jp

**Keywords:** DNA repair, IGFBP-3, oral squamous cell carcinoma, DNA-PKcs, radiosensitivity

## Abstract

Insulin-like growth factor (IGF) binding protein-3 (IGFBP-3) modulates various cell functions through IGF-dependent or independent mechanisms. However, its biological roles in the radiosensitivity of oral squamous cell carcinoma (OSCC) remain largely unknown. The purpose of this study was to determine the clinical significance and molecular mechanisms of the association between IGFBP-3 and OSCC radiosensitivity. We performed an immunohistochemical analysis of IGFBP-3 in 52 OSCC specimens from patients treated with preoperative chemoradiotherapy and surgery (phase II study). Associations between IGFBP-3 expression and clinicopathological features were also evaluated. In addition, we examined the effects of IGFBP-3 on post-X-ray irradiation radiosensitivity and DNA damage in vitro. High IGFBP-3 expression was significantly correlated with poor chemoradiotherapy responses and prognosis. With IGFBP-3 knockdown, irradiated OSCC cells exhibited significantly higher radiosensitivity compared with that of control cells. Moreover, IGFBP-3 depletion in OSCC cells reduced phosphorylation of the DNA-dependent protein kinase catalytic subunit (DNA-PKcs), which is required for DNA double-strand break repair during non-homologous end joining. These findings indicate that IGFBP-3 may have a significant role in regulating DNA repair and is be a potential biomarker for predicting clinical response to radiotherapy and prognosis in OSCC.

## 1. Introduction

Oral cancer, and especially oral squamous cell carcinoma (OSCC), is among the most common cancers worldwide [1]. Despite innovation and advances in diagnostic techniques and treatment, the prognosis for this disease has not improved [2]. As such, potential malignancy leading to therapeutic resistance has yielded unsatisfactory results.

Radiotherapy, currently a predominant component of OSCC treatment, effectively kills cancer cells by inducing DNA damage [3]. However, OSCC cells frequently develop radioresistance by activating the DNA-damage response, controlling the generation of reactive oxygen species, and modulating immune responses [4]. Although radioresistance is a major clinical obstacle for OSCC, the detailed underlying molecular mechanisms are still unknown. 

Insulin-like growth factor binding protein-3 (IGFBP-3) is one of six secreted IGFBP-family proteins that regulate insulin-like growth factor (IGF) signalling [5]; however, IGFBP-3 also has IGF-independent functions, including cellular uptake and nuclear import [6]. Moreover, many studies have examined the relationship between circulating IGFBP-3 and carcinogenesis risk or prognosis, but only a few consistent associations have been reported [7]. For example, circulating IGFBP-3 was not found associated with pancreatic, colorectal, ovarian, or breast cancer risk [8,9,10,11,12]. In lung cancer, however, circulating IGFBP-3 has an inverse correlation with cancer risk [13], while IGFBP-3 plasma levels in prostate cancer are associated with cancer incidence and survival [14,15]. Meanwhile, other studies have indicated that tumour protein and mRNA levels of IGFBP-3 may be useful prognostic markers [7]. In breast cancer and glioblastoma, high expression levels of IGFBP-3 in cancer tissues are associated with decreased survival rates [16,17]. However, there have been no reports on the association between IGFBP-3 expression and prognosis in OSCC. The role of IGFBP-3 in therapeutic resistance is also unclear. Here, we investigated IGFBP-3 expression status in biopsy specimens obtained from patients with OSCC via an immunohistochemical analysis and determined that elevated IGFBP-3 expression is significantly correlated with poor response to preoperative chemoradiotherapy and a low survival rate. Furthermore, we demonstrated that IGFBP-3 confers radioresistance by activating DNA repair after X-ray irradiation in OSCC cells. 

## 2. Results

### 2.1. Clinical Significance of IGFBP-3 Expression in Patients with OSCC

**Figure 1:**
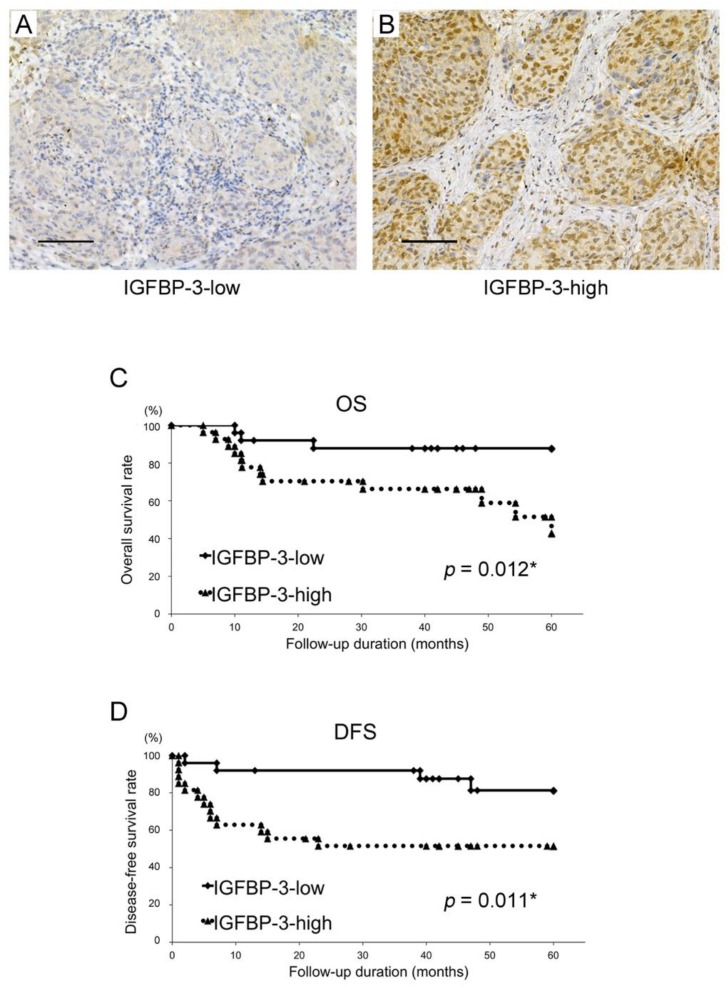
Tumour IGFBP-3 expression status affects the survival of patients with oral squamous cell carcinoma (OSCC). (**A**,**B**) Immunohistochemical staining for IGFBP-3 in OSCC biopsy specimens. Representative microscopic images show the characteristic expression status (**A** low expression; **B** high expression). Scale bar, 100 μm. (**C**,**D**) Overall survival (OS) (**C**) and disease-free survival (DFS) (**D**) of patients with OSCC based on IGFBP-3 expression status. * *p* < 0.05.

To determine the role of IGFBP-3 in OSCC, we collected biopsy specimens from 52 patients with OSCC to assess IGFBP-3 expression via immunohistochemistry. IGFBP-3 immunoreactivity was observed mainly in the nucleus and weakly in the cytoplasm of epithelial cells (Figure 1A,B). Patients were classified into low or high IGFBP-3 expression groups based on the immunostaining score for IGFBP-3. The results indicate a variety of immunostaining patterns in clinical OSCC samples, reflecting the biological properties of cancer cells. To illuminate the clinical implications of IGFBP-3 expression in OSCC, we examined correlations between IGFBP-3 expression and clinicopathological variables (Table 1). Of the 52 OSCC cases, 27 (51.9%) showed high IGFBP-3 expression and 25 (48.1%) showed low IGFBP-3 expression. As shown in Table 1, the frequency of tumours with high IGFBP-3 expression was significantly higher in patients associated with poor responses to preoperative chemoradiotherapy than in patients with good responses (*p* = 0.028). In contrast, no significant differences in IGFBP-3 expression were observed according to age, sex, primary site, clinical stage, T-stage, N-stage, or differentiation. Moreover, to examine the effect of circulating IGFBP-3, we assessed expression levels in plasma obtained from 30 patients with OSCC. Plasma IGFBP-3 levels were not associated with tissue levels (Supplementary Figure S1) and there were no significant differences in terms of clinical features and prognosis (Supplementary Tables S1 and S2).

### 2.2. Relationships between IGFBP-3 Expression and Survival

To assess the relationships between IGFBP-3 expression and survival, 52 patients were analysed for overall survival (OS) and disease-free survival (DFS) using the Kaplan–Meier method. OS and DFS within the high IGFBP-3 expression group were significantly lower than in cases with low IGFBP-3 expression (*p* = 0.012 and *p* = 0.011, respectively; Figure 1C,D). A multivariate analysis with the Cox proportional hazards regression model indicated that IGFBP-3 expression, pN-stage, and pathological response to chemoradiotherapy are significant OSCC prognostic factors for patients (Table 2).

### 2.3. Effect of IGFBP-3 on Radiosensitivity in OSCC Cells

**Figure 2:**
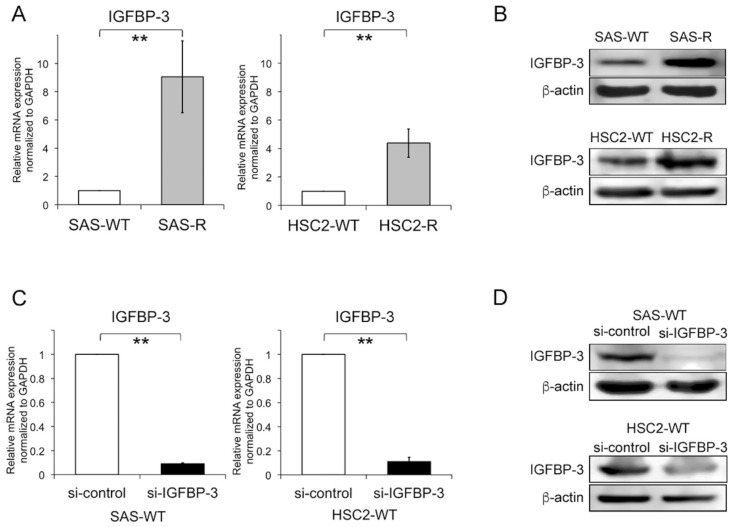

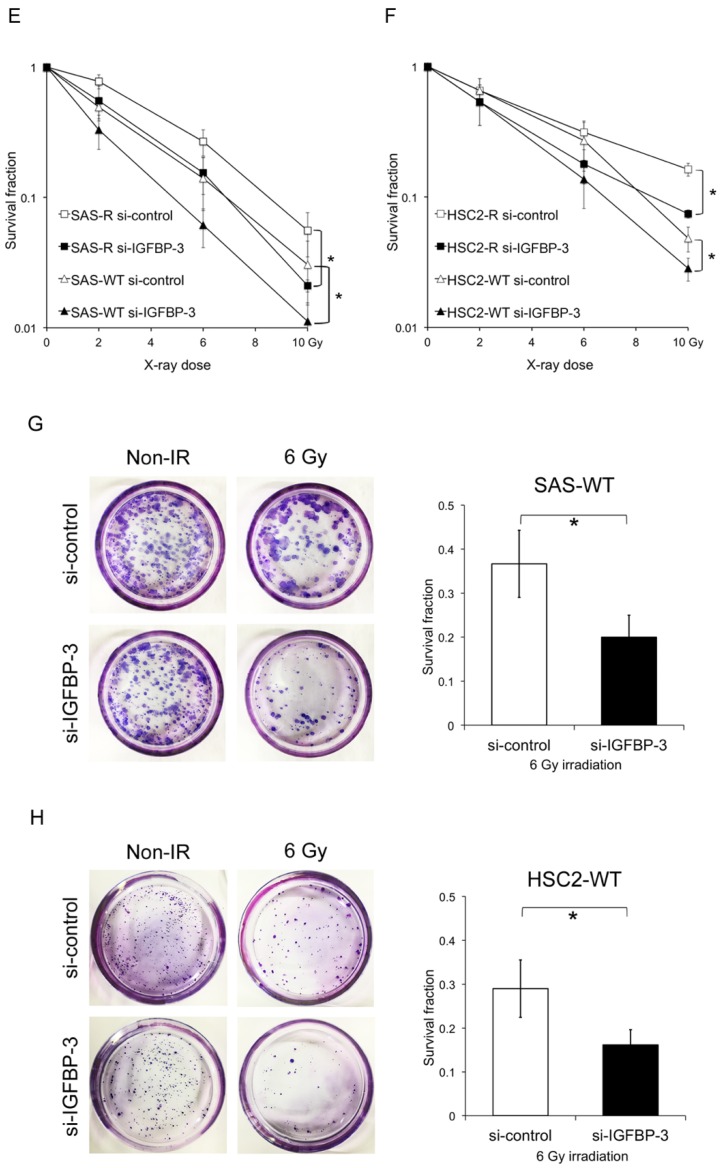
IGFBP-3 confers resistance to radiation in OSCC cells. (**A**,**B**) IGFBP-3 mRNA and protein expression levels in clinically relevant radioresistant (CRR) cells and parental cells. (**C**,**D**) IGFBP-3 mRNA and protein levels in SAS and HSC-2 cells after transfection with IGFBP-3 or control siRNA. Total RNA and whole-cell lysates were used for qRT-PCR (**A**,**C**) and Western blot analysis (**B**,**D**), respectively. (**E**,**F**) CRR and parental cells were transfected with IGFBP-3 or control siRNA and exposed to an X-ray dose of 0, 2, 6, and 10 Gy; the surviving fraction was then evaluated using a modified high-density survival (HDS) assay. (**G**,**H**) SAS and HSC-2 cells transfected with IGFBP-3 siRNA or control were exposed to 6 Gy, and then the surviving fraction was evaluated by clonogenic assays. The results are shown as the means ± SD of three independent experiments. * *p* < 0.05. ** *p* < 0.01.

To address the biological importance of IGFBP-3 in radiosensitivity, we employed clinically relevant radioresistant (CRR) OSCC cells [18]. Modified high-density survival (HDS) assays suggested that CRR cell line survival (both SAS-R and HSC2-R) was significantly enhanced compared with that of the corresponding parental cells under all exposure doses (Supplementary Figure S2). To examine IGFBP-3 expression levels in CRR cells, we performed quantitative reverse transcription PCR (qRT-PCR) and western blot analyses. The results show that IGFBP-3 expression is significantly increased at both the mRNA and protein level in SAS-R and HSC2-R cells compared with parental cells (Figure 2A,B). To further test whether IGFBP-3 affects radiosensitivity in irradiated OSCC cells, we performed modified HDS and clonogenic assays using small interfering RNA (siRNA)-mediated IGFBP-3 knockdown. Western blot and qRT-PCR analyses confirmed that IGFBP-3 was depleted at both the mRNA and protein levels (Figure 2C,D). For the modified HDS assay, each cell line was irradiated at 0, 2, 6, and 10 Gy. Irradiated CRR cells and parental cells with IGFBP-3 knockdown exhibited significantly increased radiosensitivity than that of control cells (Figure 2E,F); clonogenic assays also yielded similar results (Figure 2G,H). We then examined OSCC cellular growth activities after IGFBP-3 knockdown via cell proliferation assays and found that IGFBP-3 significantly affects cell proliferation (Supplementary Figure S3). Regarding the influence of cellular growth activity, the differences in proliferation were normalized to that of control cells in the modified HDS and clonogenic assays. Furthermore, IGFBP-3 knockdown did not affect OSCC cell sensitivity to cisplatin and 5-fluorouracil (Supplementary Figure S4). Collectively, our results suggest that IGFBP-3 confers resistance to radiation in OSCC cells.

### 2.4. Contribution of IGFBP-3 to Decreases in DNA Damage in Irradiated OSCC Cells

**Figure 3:**
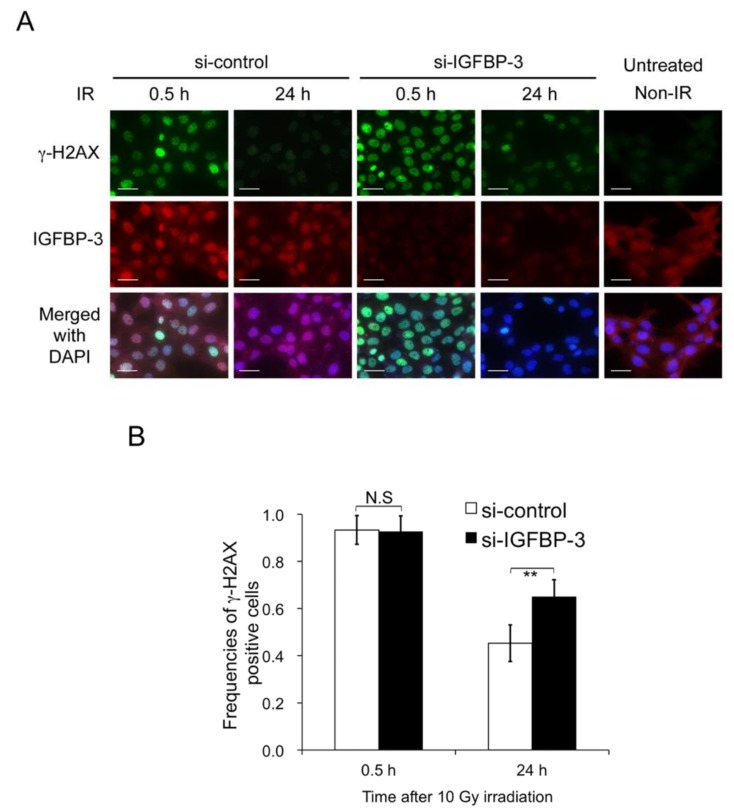
IGFBP-3 helps reduce DNA damage in irradiated OSCC cells. (**A**) Representative immunofluorescence images of γ-H2AX foci and IGFBP-3 in SAS cells after exposure to 10 Gy irradiation (IR) following transfection with control or IGFBP-3-specific siRNA. (**B**) Frequencies of γ-H2AX-positive cells at each time point after irradiation and with or without IGFBP-3 inhibition. The results are shown as the means ± SD of three independent experiments. ** *p* < 0.01. Scale bar, 50 μm.

Radiation can damage DNA stands and induce single- and double-strand breaks (DSBs). DSBs are more difficult to repair and are therefore more toxic and fatal to cancer cells [19]. To assess whether IGFBP-3 affects the number of DSBs in irradiated OSCC cells, we used γ-H2AX as a quantifiable marker after X-ray irradiation and identified cells with ≥ 10 γ-H2AX foci at 0.5 and 24 h after 10-Gy irradiation [20,21]. The frequency of γ-H2AX-positive SAS cells with IGFBP-3 knockdown (65.0 ± 7.8%) was higher than that in control cells (45.3% ± 7.2%) at 24 h (Figure 3A,B). These results suggest that IGFBP-3 helps to reduce DNA damage in irradiated OSCC cells.

### 2.5. Functional Role of IGFBP-3 in the DNA Repair of Irradiated OSCC Cells

**Figure 4:**
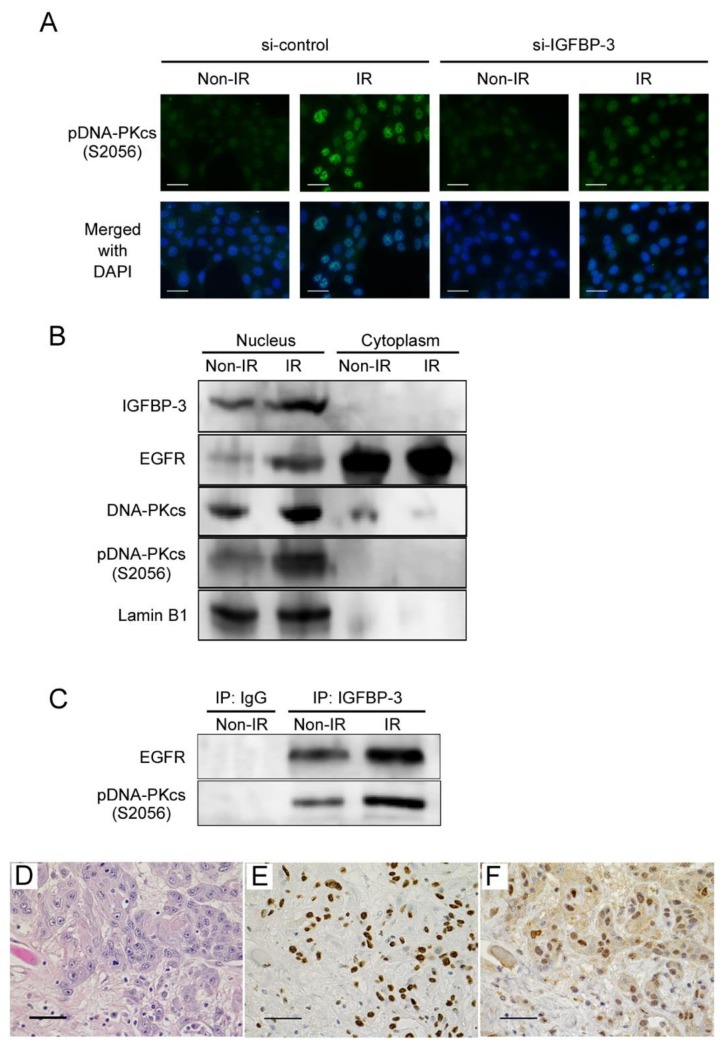
IGFBP-3 is involved in DNA-dependent protein kinase catalytic subunit (DNA-PKcs) activation by interacting directly with DNA-PKcs and epidermal growth factor receptor (EGFR) in irradiated OSCC cells. (**A**) Immunofluorescence for phospho-DNA-PKcs (S2056) in SAS cells 0.5 h after 0 or 10 Gy irradiation with or without IGFBP-3 knockdown. Scale bar, 50 μm. (**B**) Western blots of IGFBP-3, EGFR, DNA-PKcs, and phospho-DNA-PKcs protein levels in SAS cells at 0.5 h after 0 or 10 Gy irradiation. (**C**) IGFBP-3/EGFR and IGFBP-3/phospho-DNA-PKcs complex formation in SAS cells, as detected by immunoprecipitation (IP) assays. An anti-IGFBP-3 antibody was used to immunoprecipitate cell lysates, after which immunoprecipitates were subjected to immunoblotting with anti-EGFR and anti-phospho-DNA-PKcs antibodies. (**D**–**F**) Representative microscopic images of haematoxylin and eosin (**D**) and immunohistochemical staining for IGFBP-3 (**E**) and DNA-PKcs (**F**) in tumour tissues after chemoradiotherapy. Scale bar, 100 μm.

Radiation-induced DSBs can be repaired by two predominant processes: homologous recombination (HR) and non-homologous end joining (NHEJ) [22]. A previous study showed that NHEJ plays a dominant role, whereas HR has a supportive role [23]. In the NHEJ pathway, the DNA-dependent protein kinase catalytic subunit (DNA-PKcs) is essential for DSB repair [22], and the activity of this protein is associated with radioresistance [24]. Therefore, to further understand how IGFBP-3 is involved in DNA repair, we examined the activation of DNA-PKcs by assessing its phosphorylation at Ser2056 in IGFBP-3-knockdown OSCC cells after irradiation. While phosphorylated DNA-PKcs was not observed in non-irradiated cells, it was significantly enhanced in irradiated cells. IGFBP-3 knockdown markedly reduced this phosphorylation (Figure 4A), suggesting that IGFBP-3 plays an important role in DNA-PKcs activation.

### 2.6. Interactions among IGFBP-3, EGFR, and Phosphorylated DNA-PKcs in Irradiated OSCC Cells

A recent study revealed that nuclear translocation of epidermal growth factor receptor (EGFR) contributes to radioresistance by activating DNA-PKcs phosphorylation [25]. To assess the interactions among IGFBP-3, EGFR, and phosphorylated DNA-PKcs in irradiated OSCC cells, we performed a Western blot analysis and immunoprecipitation. After 0.5 h of 10 Gy radiation exposure, IGFBP-3, EGFR, and phosphorylated DNA-PKcs expression was found to be increased in the nucleus (Figure 4B). Furthermore, these complexes were increased in OSCC cells exposed to 10 Gy radiation (Figure 4C). Next, we examined the expression of IGFBP-3 and DNA-PKcs in post-operative OSCC specimens via immunohistochemistry. Interestingly, IGFBP-3 and DNA-PKcs expression levels were found to be increased in the surviving tumour cells of most cases after preoperative chemoradiotherapy (Figure 4D–F). These findings suggest that IGFBP-3 contributes to cell survival by promoting DNA repair mediated by DNA-PKcs activation in OSCC cells.

## 3. Discussion

Here, we report several meaningful findings. First, tissue but not plasma IGFBP-3 expression was found to be associated with chemoradiotherapy efficacy and prognosis in patients with OSCC. Second, IGFBP-3 levels were significantly increased in CRR OSCC cell lines. Third, IGFBP-3 knockdown increased radiosensitivity. Finally, IGFBP-3 was found to directly interact with EGFR and phosphorylated DNA-PKcs in the nucleus of irradiated OSCC cells and was associated with DNA repair. To our knowledge, there have been no other reports demonstrating the contribution of IGFBP-3 to both therapeutic resistance and poor OSCC prognosis.

Nevertheless, some studies have shown significant associations between tumour IGFBP-3 levels and cancer prognosis [16,17]. However, the clinical importance of tissue IGFBP-3 levels may differ among different tumour types [7]. In oesophageal cancer, IGFBP-3 was reported to inhibit tumour cell growth and induce apoptosis after radiotherapy [26,27]. In lung cancer, cisplatin-resistant cells exhibit reduced IGFBP3 expression [28]. In contrast, our findings indicate that IGFBP-3 can promote proliferation and increase cell survival after irradiation in OSCC. Moreover, IGFBP-3 depletion did not affect OSCC cell sensitivity to chemotherapeutic drugs. Other studies have reported that overexpression of IGFBP-3 is correlated with increased tumour size and lymph node metastasis in patients with OSCC [29,30], suggesting that IGFBP-3 may enhance malignancy in OSCC. Although tumour IGFBP-3 may contribute to tumour progression in OSCC, these discrepancies reflect the diversity of IGFBP-3 functions, including IGF-dependent and independent effects, in different types of cancers.

The association between tumour IGFBP-3 and circulating IGFBP-3, functioning as the main carrier of IGF-I and IGF-II, is yet to be fully elucidated. Moreover, the evidence for an association between circulating IGFBP-3 and either cancer risk or prognosis in different cancer types is inconsistent [8,9,10,11,12,31]. Here, we showed that plasma levels of IGFBP-3 were not associated with tissue levels of IGFBP-3, clinicopathological features, or patient prognosis in OSCC. These results indicate that unlike IGFBP-3 expression in tumour tissue, circulating IGFBP-3 may play a biologically different role.

Nuclear IGFBP-3 has been identified in human keratinocyte cell line [32] and human prostate cancer tissues [33]; indeed, this protein is known to have a functional nuclear localisation signal [34]. EGFR also has a nuclear localisation signal domain and increased nuclear localisation has been reported to enhance therapeutic resistance through associations with DNA-PKcs activation [35]. In a previous study where breast cancer cells were treated with DSB-inducing agents, Lin et al. [36] demonstrated that IGFBP-3 can form complexes with EGFR and DNA-PKcs after treatment with DNA-damaging drugs. It was also shown that upon IGFBP-3 knockdown, DNA-PKcs phosphorylation and EGFR–DNA-PKcs complex formation in the nucleus are inhibited and that NHEJ activity is also decreased. In the present study, we demonstrated that IGFBP-3 is involved in decreased DSB-DNA damage which is mediated by DNA-PKcs activation with the formation of the aforementioned complex and that high tumour IGFBP-3 levels are associated with reduced chemoradiotherapy efficacy and poor prognosis in OSCC. Taken together, these results suggest that IGFBP-3 may play a key role in DNA damage-inducing therapy, thus contributing to poor clinical outcomes.

We previously established CRR cells that continue to grow even after daily exposure to an X-ray dose of 2 Gy or more than 60 Gy, and showed that these cells efficiently repair irradiation-induced DSBs [20]. The CRR cell line is very useful for analysing the biological properties of radioresistant cells; thus, we used two CRR OSCC cell lines in this study and observed, for the first time, IGFBP-3 overexpression in these cell lines. Furthermore, it is noteworthy that blockade of IGFBP-3 augmented CRR cell radiosensitivity, suggesting that IGFBP-3 is a promising therapeutic target for overcoming OSCC radioresistance.

There are several limitations to our study. First, the obtained findings were mainly based on in vitro data, except for immunohistochemical analysis of tissues and clinical data from patients with OSCC. Second, although radiation-induced DSBs are repaired by two major pathways, namely NHEJ and HR, our analysis focused on the relationship between IGFBP-3 and the NHEJ protein DNA-PKcs. Finally, other mechanisms are involved in radioresistance, not only DNA repair, including several stress-responsive signalling pathways such as reactive oxygen species generation and activation of cytoprotective autophagy [37]. Therefore, further studies are required to confirm the effects of a combination therapy of IGFBP-3-targeting drugs and radiotherapy on in vivo models and to assess the relationship between IGFBP-3 and the HR DNA repair pathway, as well as other stress-responsive signalling pathways, in radioresistance.

## 4. Materials and Methods 

### 4.1. Clinical Samples 

For the histopathological analysis, tissue samples were obtained from 52 patients with advanced OSCC treated at Kumamoto University Hospital between October 2003 and June 2011. In addition, blood plasma samples were obtained from 30 of the 52 patients; plasma was separated by centrifugation and stored at −80 °C until subsequent analysis. All patients were treated preoperatively with a total dose of 30 Gy of concurrent chemoradiotherapy, which was followed by curative surgery as a phase II study [38,39]. Radiotherapy was administered daily at 2 Gy five times per week for 15 days. All tumours were staged according to the TNM classification of the AJCC eighth edition (2017); the degree of differentiation was determined according to the grade classification of the WHO. This study was performed in accordance with the guidelines of the Ethics Committee of Kumamoto University (project identification code: SENSHIN No.2389 and RINRI No.1427). Informed consent was obtained from all patients prior to the biopsy and operation based on the guidelines of the Kumamoto University (SENSHIN No.2389). The present study is a retrospective analysis, which does not have individual consent, however guarantees participation in the study and the opportunity to refuse participation in an opt-out format (RINRI No.1427).

### 4.2. Immunohistochemical Staining and Evaluation

Protein levels in OSCC tissue sections were analysed by immunohistochemistry, as previously described [39]. Briefly, tissue sections (4-μm thick) were deparaffinised, rehydrated using a graded alcohol series, and probed with anti-human IGFBP-3 (1:50; MAB305; R&D, Minneapolis, MN, USA) and anti-human DNA-PKcs (1:200; 3H6; Cell Signaling Technology, Danvers, MA, USA) by incubating samples at 4 °C overnight in a humid chamber. Then, sequential 60-min incubations with secondary antibodies (Dako EnVision + System-HRP Labeled Polymer; Agilent Technologies, Santa Clara, CA, USA) and visualisation with the Dako Liquid DAB + Substrate Chromogen System (Agilent Technologies) were performed. All slides were lightly counterstained with haematoxylin for 30 s prior to dehydration and mounting. Immunoreactivity for IGFBP-3 expression was evaluated by three observers blinded to patient clinical status. For each specimen, one score was assigned according to the proportion of positive cells as follows: <25%, 0 points; 25–50%, 1 point; 51–75%, 2 points; >75%, 3 points. A second score was assigned according to staining intensity, with negative staining considered 0 points, weak staining 1 point, moderate staining 2 points, and strong staining 3 points. IGFBP-3 expression scores were then calculated by multiplying the two scores. If the expression score was ≥4, the tissue was considered to have high expression. 

### 4.3. Cell lines and Culture

We obtained human OSCC cell lines derived from primary tumours, namely SAS and HSC-2, from the Japanese Collection of Research Bioresources Bank of the National Institutes of Biomedical Innovation, Health and Nutrition (Osaka, Japan). In the present study, HSC-2 cells were simply designated HSC2. The CRR cell lines SAS-R and HSC2-R were derived from SAS and HSC2 cells, respectively, by exposing cells to gradually increasing X-ray doses [18,20]. These cells continued to proliferate with daily 2-Gy irradiation for more than 30 days in vitro and were resistant to irradiation. Cells were cultured in DMEM supplemented with 10% FBS in a humidified atmosphere of 5% CO_2_ at 37 °C. 

### 4.4. Irradiation 

Irradiation doses of 2, 6, and 10 Gy were administered with a 150-KVp X-ray generator (MBR-1520R; Hitachi, Tokyo, Japan) with a total filtration of 0.5-mm aluminium plus a 0.1-mm copper filter. The dose rate (1.01 Gy·min^−1^) was measured with a thimble ionisation chamber (IC 17A; Far West Technology, Goleta, CA, USA).

### 4.5. siRNA Transfection

Twenty-four hours prior to transfection, SAS and HSC2 cells were diluted in fresh medium without antibiotics. They were then transfected with IGFBP-3-specific siRNA (20 nM; Stealth siRNA; Invitrogen, Carlsbad, CA, USA) using Lipofectamine RNAi MAX (Invitrogen), as per the manufacturer’s instructions. Cells were harvested 48 h post-transfection.

### 4.6. RNA Isolation and qRT-PCR 

Total RNA was isolated using the mirVana miRNA Isolation Kit (Life Technologies, Carlsbad, CA, USA) and then reverse-transcribed to cDNA using the ReverTra Ace qPCR RT Kit (Toyobo, Osaka, Japan). qPCR was performed using Thunderbird SYBR qPCR Mix (Toyobo) on a Light Cycler 1.5 (Roche, Basel, Switzerland). We used the comparative Ct (DDCt) method to determine fold-changes in expression levels using glyceraldehyde-3-phosphate dehydrogenase (*GAPDH*) as a housekeeping gene. Each sample was run in triplicate. The following primers were used: IGFBP-3 forward, 5′-CAAGCGGGAGACAGAA-3′ and reverse, 5′-GGACTCAGCACATTGAGGAACTT-3′; and GAPDH forward, 5′-CTGGGCTACACTGAGC-3′ and reverse, 5′-AAGTGGTCGTTGAGGG-3′. Cycling conditions were as follows: initial denaturation at 98 °C for 5 min, followed by 45 cycles of 98 °C for 15 s, 58 °C for 30 s, and 72 °C for 60 s. PCR data were collected from three independent experiments.

### 4.7. HDS Assays 

HDS assays were performed as described by Kuwahara et al. [21,40]. Exponentially growing cells (5 × 10^5^) were seeded in 60-mm tissue culture dishes (AGG Inc., Tokyo, Japan) and incubated in DMEM supplemented with 10% FBS for 48 h. Cells were transfected with scrambled siRNA and then exposed to 2, 6, and 10 Gy. After 72 h, 10% of the cells in each flask were seeded in a new 60-mm culture dish and incubated for 72 h. Total cell numbers in each culture dish were determined using Trypan blue exclusion, and cell survival was plotted. 

### 4.8. Clonogenic Assays

After irradiating siRNA-transfected cells with 6 Gy, the cells (1 × 10^3^) were seeded in a 60-mm culture dish coated with gelatine (AGG Inc.) and cultured in DMEM supplemented with 10% FBS for 10 days. Next, the cells were fixed with 99.5% methanol and stained with Giemsa solution (Wako, Osaka, Japan). 

### 4.9. Immunofluorescent Staining and Evaluation 

Cells (2 × 10^4^) were transfected with siRNA, seeded on glass slides (Merck Millipore, Burlingame, CA, USA), cultured in DMEM supplemented with 10% FBS for 24 h, and irradiated with 10 Gy. After 0.5 h (for γ-H2AX and pDNA-PKcs) or 24 h (for γ-H2AX), cells were fixed for 30 min with 4% paraformaldehyde in PBS. Then, cells were washed with PBS-T for 5 min and blocked with PBS-T containing 5% BSA for 5 min on ice. Next, cells were incubated for 2 h at room temperature with primary antibodies against phosphorylated histone H2AX (γ-H2AX; 1:400; cat# 613401; BioLegend, San Diego, CA, USA), IGFBP-3 (1:50; H-98; Santa Cruz Biotechnology, Dallas, TX, USA), and S2056-phopho-DNA-PKcs (1:200; ab18192; Abcam, Cambridge, UK) in PBS-T with 1% BSA. Glass slides were then washed with PBS-T three times (5 min each), and cells were incubated at room temperature for 1.5 h with secondary antibodies (Alexa Fluor 488 and 594 donkey anti-rabbit and/or anti-mouse IgG; Life Technologies) in PBS-T with 1% BSA. After washing with PBS, cells were counterstained and mounted with Vectashield (Vector Laboratories, Inc., Burlingame, CA, USA) and then imaged using a fluorescent microscope (BZ-X700; Keyence, Osaka, Japan). Cells were considered γ-H2AX-positive if they had >10 foci per nucleus [41].

### 4.10. Western Blot Analysis

Whole-cell and nuclear proteins (Minute Cytoplasmic and Nuclear Extraction Kits; Invent Biotechnologies, Inc., Eden Prairie, MN, USA) were separated by 5% or 12.5% SDS-PAGE, transferred to nitrocellulose membranes, and probed with antibodies specific for IGFBP-3 (1:200; H-98; Santa Cruz Biotechnology), epidermal growth factor receptor (EGFR) (1:1000; D38B1; Cell Signaling Technology), DNA-PKcs (1:1000; 3H6; Cell Signaling Technology), phospho-DNA-PKcs (S2056) (1:400; ab18192; Abcam), lamin B1 (1:1000; Abcam), and β-actin (1:5000; Sigma-Aldrich, St Louis, MO, USA). After incubating the membranes overnight at 4 °C, they were washed, incubated with the appropriate horseradish peroxidase-conjugated secondary antibodies, and developed using the ECL prime detection kit (GE Healthcare, Chicago, IL, USA). The emitted light was measured using the C-DiGit blot scanner and the images were analyzed using Image studio for C-Digit (LI-COR Biosciences, Lincoln, NE, USA). Densitometry readings and the whole blot images are provided in the Supplementary Materials.

### 4.11. Immunoprecipitation

Proteins were extracted in RIPA buffer (s25 mM tris-HCl (pH 7.6), 150 mM NaCl, 1% NP-40, 1% sodium deoxycholate, and 0.1% SDS) with a protease inhibitor cocktail (Roche Diagnostics). Total purified proteins were incubated with 5 mg of antibody specific for IGFBP-3 (Santa Cruz Biotechnology) to form immunocomplexes, which were precipitated with protein A-Sepharose (GE Healthcare). Finally, immunocomplexes were subjected to Western blotting.

### 4.12. Cell Proliferation Assays

Cell proliferation was determined using the cell proliferation reagent WST-8 (Cell Counting Kit-8; Dojindo, Kumamoto, Japan). In brief, for each condition, 3 × 10^3^ cells were seeded in 96-well plates in 100 mL of medium in quadruplicate. At 24, 48, and 72 h, WST-8 reagent was added at a 1:10 dilution and plates were incubated for an additional 1 h at 37 °C. The absorbance was measured at 690/480 nm. Data were collected from three independent experiments.

### 4.13. Enzyme-Linked Immunosorbent Assay (ELISA) for Plasma IGFBP-3

Plasma IGFBP-3 levels were measured using a Human IGFBP-3 Quantikine ELISA Kit (R&D Systems) according to the manufacturer’s instructions.

### 4.14. Statistical Analysis

Differences in mean values between two groups were analysed using Student’s *t*-tests, while differences in mean values among multiple groups were analysed by one-way ANOVA with the Bonferroni/Dunn test. For small sample sizes showing normal distribution, Student’s *t*-tests were performed. For tissue specimens, the χ2-test was used to determine associations between IGFBP-3 expression and clinical parameters. A Fisher’s exact test was used when one or more cells had expected values <5. A survival analysis was performed using the Kaplan–Meier method. The log-rank test was used to determine correlations between IGFBP-3 expression and patient survival. A multivariate survival analysis was performed using the Cox regression model to determine the effects of IGFBP-3 expression on OS and DFS. All *p*-values were based on two-tailed statistical analyses; *p*-values < 0.05 were considered statistically significant. All analyses were performed with JMP 9 software (SAS Institute Inc, Cary, NC, USA).

## 5. Conclusions

Here, we highlighted the potential importance of IGFBP-3 in radioresistant OSCC. Our findings indicate that tumour IGFBP-3 expression can be exploited to predict responses to chemoradiotherapy and that IGFBP-3 may represent a novel prognostic factor. Therapies targeting IGFBP-3-dependent DNA repair combined with radiotherapy also represent a promising new approach for overcoming OSCC radioresistance.

## Figures and Tables

**Table 1:** Correlation between IGFBP-3 expression and clinicopathological factors in 52 patients with oral squamous cell carcinoma (OSCC).

Characteristic	Total	IGFBP-3 Status		*p*-Value
		High Expression	Low Expression	
		*n* (%)	*n* (%)	
	52	27 (51.9)	25 (48.1)	
Age, years				
Median	66.5	67.3	65.6	
Range	30–85	30–85	47–84	
≤65	23	9 (39.1)	14 (60.9)	0.100
>65	29	18 (62.1)	11 (37.9)	
Sex				
Male	31	13 (41.9)	18 (58.1)	0.080
Female	21	14 (66.7)	7 (33.3)	
Primary site				
Tongue	16	9 (56.3)	7 (43.7)	0.570
Mandible gingiva	15	8 (53.3)	7 (46.7)	
Maxilla gingiva	7	5 (71.4)	2 (28.6)	
Buccal mucosa	10	4 (40.0)	6 (60.0)	
Oral floor	4	1 (25.0)	3 (75.0)	
Clinical stage				
III	18	10 (55.6)	8 (44.4)	
IV	34	17 (50.0)	17 (50.0)	
pT-stage				
T2	10	4 (40.4)	6 (60.0)	0.545
T3	24	12 (50.0)	12 (50.0)	
T4	18	11 (61.1)	7 (38.9)	
pN-stage				
N0	31	18 (58.1)	13 (41.9)	0.282
≥N1	21	9 (42.9)	12 (57.1)	
Differentiation				
Well	40	21 (55.3)	19 (44.7)	0.879
Moderate, poor	12	6 (50.0)	6 (50.0)	
Pathological response				
Grade 0, I, II	29	19 (65.5)	10 (34.5)	0.028 *
Grade III, IV	23	8 (34.8)	15 (65.2)	

A chi-square test was used to examine the correlation between IGFBP3 expression and clinicopathological factors in 52 patients with OSCC. * *p* < 0.05.

**Table 2:** Multivariate analysis of prognostic factors in patients with OSCC based on the Cox proportional hazards regression model and IGFBP-3 expression.

Characteristic	Assigned Score	OS		DFS	
		Hazard Ratio (95% CI)	*p*-Value	Hazard Ratio (95% CI)	*p*-Value
IGFBP-3 expression					
Low	0	3.783 (1.148–17.11)	0.028 *	3.878 (1.294–14.35)	0.014 *
High	1				
pN-stage					
N0	0	2.951 (1.033–8.598)	0.043 *	3.107 (1.136–8.641)	0.028 *
≥N1	1				
Pathological response					
Grade 0, I, II	0	0.087 (0.005–0.446)	0.001 **	0.135 (0.021–0.495)	0.001 **
Grade III, IV	1				

CI, confidence interval; DFS, disease-free survival; OS, overall survival. * *p* < 0.05, ** *p* < 0.01.

## Supplementary Materials

The following are available online at https://www.mdpi.com/2072-6694/12/2/494/s1, Figure S1: Relationship between tumour IGFBP-3 levels and plasma IGFBP-3 levels in patients with oral squamous cell carcinoma (OSCC), Figure S2: Differences in radiosensitivity between clinically relevant radioresistant (CRR) cells and parental cells, Figure S3: IGFBP-3 knockdown inhibits cell proliferation in OSCC cells, Figure S4, IGFBP-3 knockdown does not affect OSCC cell sensitivity to chemotherapeutic drugs, Table S1: Correlation between plasma IGFBP3 levels and clinicopathological factors in 30 patients with oral squamous cell carcinoma (OSCC), Table S2: Prognostic analysis of plasma IGFBP3 levels by the Cox proportional hazards regression model.
